# Microdissection of Distinct Morphological Regions Within Uveal Melanomas Identifies Novel Drug Targets

**DOI:** 10.3390/cancers16244152

**Published:** 2024-12-13

**Authors:** Elsa Toumi, Luke B. Hesson, Vivian Lin, Dale Wright, Elektra Hajdu, Li-Anne S. Lim, Michael Giblin, Fanfan Zhou, Alexandra Hoffmeister, Farida Zabih, Adrian T. Fung, R. Max Conway, Svetlana Cherepanoff

**Affiliations:** 1 Department of Ophthalmology, University Hospital of Nice, 06000 Nice, France; elsatoumi@hotmail.fr; 2 SydPath, St Vincent’s Hospital Sydney, Darlinghurst, NSW 2010, Australia; ahoff82@gmail.com (A.H.);; 3 Medicine & Health, UNSW Sydney, Randwick, NSW 2031, Australia; l.hesson@unsw.edu.au (L.B.H.); vivian.lin.vl@gmail.com (V.L.); 4 Kinghorn Centre for Clinical Genomics, Garvan Institute of Medical Research, Darlinghurst, NSW 2010, Australia; e.hajdu@garvan.org.au; 5 Department of Molecular Genetics, Douglass Hanly Moir Pathology, Macquarie Park, NSW 2113, Australia; 6 Department of Cytogenetics, Sydney Genome Diagnostics, The Children’s Hospital at Westmead, Westmead, NSW 2145, Australia; dale.wright@health.nsw.gov.au; 7 Discipline of Paediatrics & Child Health, Faculty of Medicine and Health, The University of Sydney, Sydney, NSW 2050, Australia; 8 Ocular Oncology Unit, Sydney Eye Hospital, Sydney, NSW 2000, Australia; limlianne@gmail.com (L.-A.S.L.); emgiblin@ozemail.com.au (M.G.); drmaxconway@gmail.com (R.M.C.); 9 Sydney Pharmacy School, The University of Sydney, Sydney, NSW 2050, Australia; fanfan.zhou@sydney.edu.au; 10 Westmead and Central Clinical Schools, Specialty of Ophthalmology and Eye Health, The University of Sydney, Sydney, NSW 2050, Australia; adrian.fung@sydney.edu.au; 11 Department of Ophthalmology, Faculty of Medicine, Health and Human Sciences, Macquarie University, Sydney, NSW 2113, Australia; 12 School of Medicine, University of Notre Dame, Sydney Campus, Darlinghurst, NSW 2010, Australia

**Keywords:** uveal melanomas, tumour heterogeneity, microdissection, sequencing, cancer

## Abstract

**Background/Objectives**: Uveal melanomas (UMs) are rare but often deadly malignancies that urgently require viable treatment options. UMs often exhibit tumour heterogeneity, with macroscopic and microscopic differences in morphology between different regions of the same tumour. However, to date, the clinical significance of this and how it may help guide personalised therapy have not been realised. **Methods**: Using targeted DNA and RNA sequencing of a small case series of large, high-risk primary UMs, we explored whether morphologically distinct regions of the same tumour were associated with distinct molecular profiles. **Results**: In four of the seven tumours analysed, we detected different sets of genetic variants following the separate analysis of microdissected melanotic and amelanotic regions of the same tumour. These included a *MET* exon 14 skipping RNA transcript that predicts sensitivity to crizotinib and variants in other genes that are important in active clinical trials for patients with UM and advanced solid tumours. The integration of TCGA data also identified recurrent mutational events in genes that were not previously implicated in UM development (*FANCA*, *SLX4*, *BRCA2*, and *ATRX*). **Conclusions**: Our findings show that the molecular analysis of spatially separated and morphologically distinct regions of the same tumour may yield additional, therapeutically relevant genetic variants in uveal melanomas and have implications for the future molecular testing of UMs to identify targeted therapies.

## Simple Summary

Separately sequencing morphologically distinct regions within large, high-risk uveal melanomas provides deeper insights into tumour biology and identifies additional targets for therapy. Uveal melanomas are rare intraocular malignancies that exhibit significant heterogeneity, with macroscopic and microscopic differences between tumour regions. This study used microdissection and targeted sequencing to analyse melanotic and amelanotic regions within the same tumours. In four of seven cases, distinct molecular profiles were identified, including a MET exon 14 skipping transcript predictive of sensitivity to crizotinib. Additional variants were detected in genes relevant to active clinical trials. These findings highlight the importance of analysing spatially distinct tumour regions to identify actionable genetic alterations that may be missed in whole tumour aggregate analyses. This approach has implications for the molecular testing of uveal melanomas, guiding personalised therapies, and expanding eligibility for clinical trials in patients with advanced disease.

## 1. Introduction

Uveal melanoma (UM) is the most common primary intraocular malignancy among adults [1]. Despite their shared embryonic neural crest origin, UM and mucocutaneous melanoma differ substantially in their aetiology, molecular profile, and clinical course [2,3]. The advent of targeted therapies and immunotherapy has markedly improved the survival of patients with metastatic cutaneous melanoma in the past decade [4]. There has been no substantial improvement in survival for metastatic UM for over 30 years [5]; although, clinical trials of new drugs are ongoing [6]. Despite effective primary treatment, nearly 50% of UM patients develop metastases. Due to a lack of available therapy, 20–30% of patients diagnosed with a UM die of systemic metastases within 5 years of diagnosis and 45% within 15 years [7,8,9]. Reducing this stark disparity in survival rates is a priority in ocular oncology.

The risk of UM metastasis can be accurately predicted using the American Joint Committee on Cancer (AJCC) TNM staging system [10,11,12,13], as well as established clinical and histopathologic features. Risk prognostication can be further refined by combining clinical, histopathological, and copy number changes in chromosomes 3 and 8q [14,15]. Metastatic risk can be also stratified based on four molecular categories using data from The Cancer Genome Atlas (TCGA) [3], confirmed in larger cohorts [16,17]. Unlike mucocutaneous melanoma or other solid tumours, somatic variants in the UM driver genes (GNAQ, GNA11, EIF1AX, SF3B1, PLCB4, CYSLTR2, and BAP1) are not molecular targets of currently approved therapies. Trials of targeted cancer therapies in UM patients have, thus far, shown disappointing results [18,19,20,21], as have trials of immunotherapy [22,23].

We hypothesised that identifying actionable targets in UM may require a more nuanced approach. Tumour heterogeneity is a well-documented phenomenon, whereby populations of cells within the same primary tumour show different macroscopic, histological, or molecular features. UM tumour heterogeneity has been demonstrated in multiple studies using fluorescence in situ hybridisation, gene expression profiling, single-cell sequencing, and epigenetic analyses [24,25,26,27,28,29,30,31,32]. In our experience with UMs observed over time, different tumour regions can often be discernible by way of pigmentation, growth pattern, growth rate, and echogenicity. In larger UMs, macroscopic photography in the pathology laboratory can reveal two or more conspicuous morphological regions. Furthermore, corresponding histological differences may also be observed by way of cell type, extravascular matrix pattern, growth pattern (solid versus diffuse), and differences in tumour-infiltrating macrophages and lymphocytes.

In this study, we analysed different morphological regions of the same tumour to investigate whether they are associated with different molecular features and whether this can identify additional therapeutically relevant sequence variants.

## 2. Materials and Methods

### 2.1. Study Cohort Selection Criteria

Cases included in this series met the following criteria: (i) large choroidal or ciliochoroidal tumour (at least TNM pT3, AJCC 8th edition [10]); (ii) tumour heterogeneity as defined by at least two morphologically conspicuous regions on macroscopic assessment; (iii) high metastatic risk, as determined by the minimal or absent nuclear expression of BAP1 immunohistochemistry (IHC) and monosomy or chromosome 3 or loss of 3p by CGH/SNP chromosome microarray; (iv) no previous treatment; and (v) tumour uncomplicated by infarction, necrosis, or haemorrhage.

### 2.2. Patient and Tumour Characteristics

Patient and tumour variables determined at each patient’s pre-enucleation examination were age, intraocular tumour location (choroidal or cilio-choroidal), largest basal diameter, and maximal thickness of the tumour in millimetres, as measured by B-scan ultrasonography. Post-enucleation, tumour basal diameter and thickness were confirmed by macrophotography in the laboratory (see below), as was the presence of heterogeneous tumour clones. The presence or absence of extrascleral extension and vortex vein involvement were determined at the time of specimen grossing and macrophotography. Each pathological parameter was confirmed using microscopic examination. One of the patients (Case 3) had a history of familial breast cancer and known germline BRCA2 mutation; none of the other patients had a known cancer predisposition syndrome.

### 2.3. Enucleation Grossing

Enucleated eyes were oriented according to established protocols [33]. Transillumination was performed to localise each tumour, and the eye was opened to best demonstrate the tumour mass within the pupil to the optic nerve block and to ensure that the tumour was included in the first calotte removed. The location, thickness, and basal diameter of the tumour were documented, including the involvement of the ciliary body and other structures and the presence of extrascleral extension or vortex vein involvement. A fresh tumour sample was harvested from the first calotte and submitted for CGH/SNP chromosome microarray analysis in RPMI medium. The open eye was then fixed for 48 h in 10% neutral buffered formalin (NBF). Eyes were submerged in 70% alcohol before macrophotography (Olympus SZ61 dissecting microscope, DP22 camera and cellSense software v2.0; Olympus Corporation, Notting Hill, VIC, Australia) to confirm tumour location, size, and the presence of distinct clones. The second calotte was removed after fixation, and the entire eye was subjected to paraffin embedding, sectioning, H&E staining, and microscopic examination in the following blocks: (i) vortex veins, (ii) optic nerve margin, (iii) pupil to optic nerve block, (iv) calotte #1, and (v) calotte #2.

### 2.4. Histology and Immunohistochemistry

Tissue cassettes containing tumours were processed on the Tissue Tek VIP 3 (Leica Biosystems) overnight cycle (2 h additional 10% NBF, followed by progressive dehydration with alcohol, xylol clearing, and paraplast (paraffin wax) embedding. Tissue blocks were then sectioned at 3 μm on a Leica RM2235 rotary microtome (Leica Biosystems, Wetzlar, Germany) onto frosted glass slides (Trajan Scientific and Medical, Bethel, CT, USA), dried, and stained with haematoxylin and eosin on a Tissue Tek Prisma Plus automated slide stainer (Sakura Finetek, Torrance, CA, USA), and cover slipped on an automated Tissue Tek Glas cover slipper (Sakura Finetek, USA).

Melanocytic differentiation was confirmed by haematoxylin and eosin (H&E) staining and MART1 immunohistochemistry.

Histological tumour characteristics were reported in a synoptic format according to the AJCC 8th edition [34] criteria. A set panel of immunohistochemical studies was performed as part of the standard UM protocol. Paraffin-embedded tissues were cut at 3 μm onto charged glass slides (Trajan Scientific and Medical). Immunohistochemistry was performed using the Ventana BenchMark Ultra platform (Roche Diagnostics, Basel, Switzerland). The primary antibodies, antigen retrieval, and incubation time are summarised in Supplementary Table S1. Positive and negative control tissue (informed by the datasheet of each primary antibody) was included on each slide submitted for immunohistochemistry. BAP1 immunohistochemistry was interpreted as “aberrant” when there was minimal or absent staining in tumour nuclei.

### 2.5. Comparative Genomic Hybridisation (CGH)/Single Nucleotide Polymorphism (SNP) Chromosome Microarrays

For tumour samples received prior to mid-2020, a CGH/SNP chromosome microarray was performed at SydPath. Genomic DNA from fresh tumour samples was extracted using the Gentra Puregene kit (Qiagen, Hilden, Germany) using the manual DNA extraction method with overnight proteinase-K + buffer ALT (lysis buffer) tissue digestion at 56 °C, followed by DNA purification and precipitation. CGH/SNP chromosome microarray was performed using the Applied Biosystems Cytoscan 750K assay (Thermo Fisher Scientific, Waltham, MA, USA) according to the manufacturer’s protocol. Microarray data were analysed as a CEL file using Chromosome Analysis Suite v3.1 (Thermo Fisher Scientific), with an average copy number resolution of ~0.1 Mb.

CGH/SNP chromosome microarray of tumour samples received after mid-2020 were tested at The Children’s Hospital at Westmead. Genomic DNA was extracted from fresh tissue samples using the Wizard Genomic DNA purification kit (Promega, Madison, WI, USA), but with slight modifications that involved overnight proteinase-K (Qiagen) buffered tissue digestion at 56 °C, followed by DNA purification and precipitation. SNP microarray analysis was performed using the CytoSNP 850K Beadchip v1.2 (Illumina, San Diego, CA, USA) with microarray data analysed using BlueFuse Multi v4.5 (Illumina) using a mean effective resolution of ~0.05 Mb for copy number and ~5 Mb for the copy-neutral loss of heterozygosity.

### 2.6. Reporting Criteria for CGH/SNP Chromosome Microarray Abnormalities

Similar reporting criteria were applied for both CytoScan-750K and CytoSNP-850K BeadChip assays (Illumina). Chromosome copy number abnormalities were reported when involving non-mosaic whole chromosomes (gain, loss, or copy-neutral loss of heterozygosity [CNLOH]) or segmental chromosome abnormalities (defined as >5 Mb in size). Copy number imbalances < 5 Mb were reported in overlapping tumour-relevant consensus cancer genes as per the Catalogue of Somatic Mutations in Cancer (COSMIC). Mosaic or subclonal copy number imbalances and CNLOH were reported when present in >30% of cells, as determined by assessment of the Log R and altered allele and B-allele frequency plots of SNP probes [35].

### 2.7. Extraction of DNA and RNA from FFPE Tissue

Macrodissection was performed from a minimum of 12 µm tissue, and DNA and RNA were extracted using the AllPrep DNA/RNA FFPE Kit (Qiagen, Hilden, Germany) and QIAcube instrument (Qiagen, Hilden, Germany). DNA quantity was determined using a Qubit dsDNA HS Assay kit (Thermo Fisher Scientific) and a Qubit 4 Fluorometer (Thermo Fisher Scientific).

### 2.8. Targeted Sequencing of DNA and RNA

Libraries were built using Oncomine Comprehensive Assay v3 (OCAv3, Thermo Fisher Scientific), which is an amplicon-based panel targeting the DNA or RNA of a total of 161 genes relevant to solid tumours (see Supplementary Table S2 for a full description of the targeted genes and gene fusion events). Briefly, DNA (20 ng) was subjected to multiplex polymerase chain reaction (PCR) amplification using an Ion AmpliSeq Library Plus Kit (Thermo Fisher Scientific) and DNA Oncomine Comprehensive Panel v3M (Thermo Fisher Scientific). RNA (40 ng) was subjected to reverse transcription using a SuperScript IV VILO Master Mix (Thermo Fisher Scientific), followed by library generation using an Ion AmpliSeq Library Plus Kit (Thermo Fisher Scientific) and RNA Oncomine Comprehensive Panel v3M (Thermo Fisher Scientific). The PCR products were ligated to the IonCode Adapter (Thermo Fisher Scientific) and purified using Agencourt AMPure XP beads (Beckman Coulter, Brea, CA, USA). DNA and RNA libraries were quantified on a QuantStudio 7 real-time thermocycler (Thermo Fisher Scientific) using an Ion Library TaqMan Quantitation Kit (Thermo Fisher Scientific). Each purified library sample (50 pM) was pooled and prepared for sequencing on an IonChef instrument (Thermo Fisher Scientific) using an Ion S5 Chef Kit and Ion 540 Chip (Thermo Fisher Scientific). Ion semiconductor sequencing was performed using an S5 GeneStudio (400 flows or equivalent to ~200 bp, Thermo Fischer Scientific). Sequencing was performed to a mean coverage of ≥1000× for DNA targets and ≥0.5 M total mapped reads for RNA. DNA and RNA sequencing quality was assessed using Torrent Suite v5.10.1 (Thermo Fisher Scientific).

### 2.9. Variant Calling, Annotation, and Curation

Reads were aligned to the hg19 human reference genome sequence using Ion Reporter™ Software v.5.8 (Thermo Fisher Scientific). Variant calling was performed using Ion Reporter™ with a variant allele fraction (VAF) threshold of 5%. All variants were annotated manually using Alamut Visual v2.15 software (Interactive Biosoftware, Sofia Genetics, Lausanne, Switzerland) with reference to the RefSeq transcripts provided in Supplementary Tables S2 and S3. The clinical actionability of variants was curated according to standards and guidelines described by the Association for Molecular Pathology, American Society of Clinical Oncology, and College of American Pathologists (AMP/ASCO/CAP) [36]. Allele frequencies were determined using gnomAD (v3.1.2). Variants identified as Tier IV (AMP/ASCO/CAP criteria) or with an allele frequency ≥ 0.01 in any population were excluded from the study.

## 3. Results

### 3.1. Tissue Heterogeneity in Large UMs

Of the 46 enucleated UMs submitted to our diagnostic pathology laboratory between 2017 and 2020, 39 were primary referrals, and 7 were second opinion consultations from outside laboratories. Of the primary referrals, seven met the inclusion criteria for the current study (see the study cohort selection criteria in Section 2). Six of the seven UMs showed ciliary body involvement. None of the patients exhibited extraocular extension. At the time of writing, three patients developed metastases, two died of metastatic disease, and one received post-surgical fotemustine treatment. The patient and tumour characteristics are summarised in Table 1.

**Figure 1:**
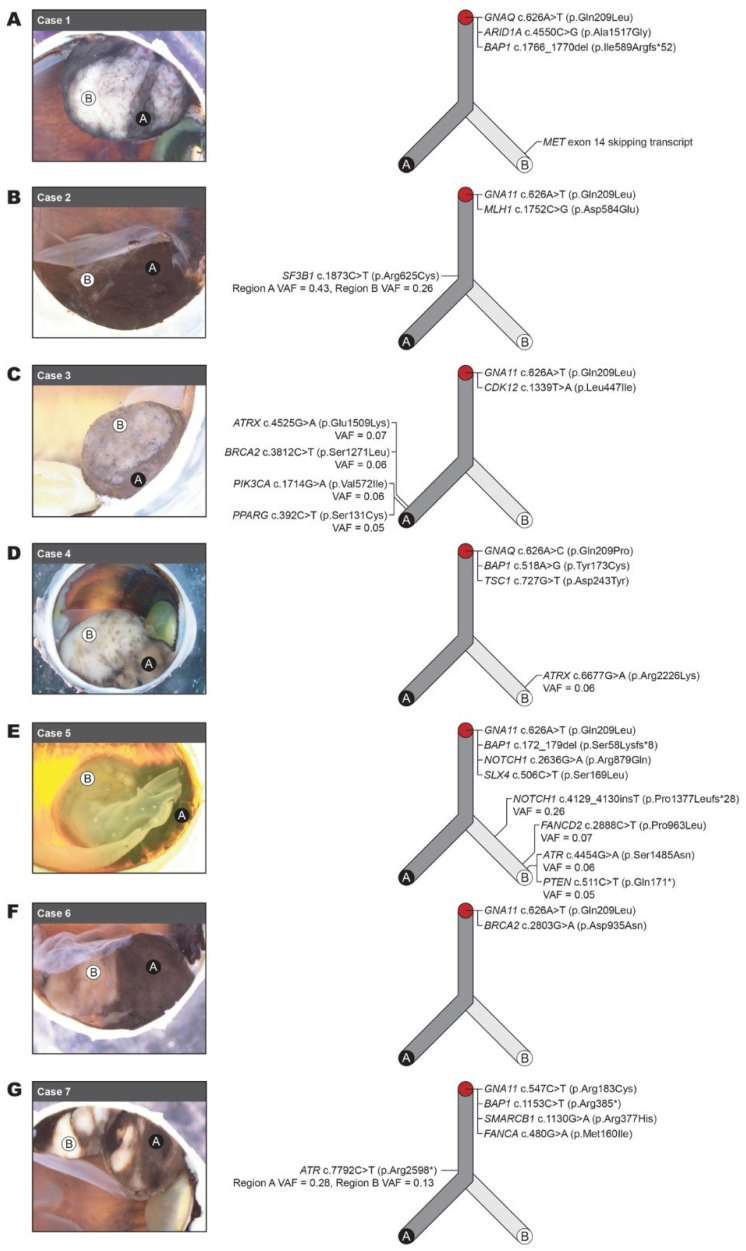
Molecular heterogeneity between morphologically distinct regions of the same tumour. Photographs on the left of panels (**A**–**G**) show the macroscopic appearance of each tumour investigated (Cases 1 to 7). Region A indicates the highly pigmented region analysed; Region B indicates the less pigmented region analysed. Schematic on the right of each panel represents a hypothesised phylogenetic tree of the molecular events in each tumour with dark grey representing highly pigmented tissue (Region A) and light grey representing less pigmented tissue (Region B). Initiating events found in the majority (≥60%) of tumour cells based on VAF are indicated at the top (red circle). Variants specific to Region A or B are indicated in respective branches with later molecular events (lower VAF) appearing towards the end of the branch. The VAF for subclonal variants (<60%) is indicated.

Upon macroscopic assessment, at least two regions exhibiting clear pigmentation differences were identified in each tumour (Figure 1), hereafter referred to as Region A (melanotic) and Region B (amelanotic).

Histological examination also revealed microscopic differences between these regions. Specifically, the tumour growth pattern was “solid” in Region B from all tumours but a mixture of “solid and diffuse” in Region A from two tumours, and microvascular density was lower in Region B when compared with Region A in five of seven tumours (Table 2).

Differences were also observed in the extravascular matrix pattern, tumour-infiltrating lymphocytes, and tumour-infiltrating CD68+/CD163+ macrophage subsets; although, these differences were not consistent between Regions A and B across the tumours.

### 3.2. Molecular Features of High Metastatic Risk in All Tumours Examined

Loss of *BAP1* is associated with a high risk of metastasis in UMs. We investigated BAP1 protein expression by immunohistochemistry, as well as *BAP1* copy number and mutation status. Copy number analysis by comparative genomic hybridisation/single nucleotide polymorphism (CGH/SNP) chromosome microarrays was performed on bulk tumour DNA extracts only (i.e., without microdissection), in accordance with routine diagnostic testing. We observed aberrant BAP1 protein levels with immunohistochemistry (IHC) in all seven tumours (Table 3). The biallelic loss of *BAP1* was confirmed in four of the seven tumours by a combination of monosomy 3 or loss of chromosome 3p and sequence variants; whereas, in three tumours, only monosomy 3 or loss of chromosome 3p was detected. In addition, chromosome 8q gain, another molecular feature of high metastatic risk, was observed in five tumours (Table 3).

Taken together, these findings identify poor prognostic features indicative of high metastatic risk in all seven tumours.

### 3.3. Molecular Heterogeneity Revealed by Microdissection and Targeted Sequencing

Regions A and B were separately microdissected (Supplementary Figure S1) and sequenced using a gene panel targeting a total of 161 genes at the DNA and RNA levels, including the known UM driver genes *GNAQ*, *GNA11*, *SF3B1*, and *BAP1* (Table 4).

All seven tumours contained a hotspot variant in either *GNAQ* (two tumours) or *GNA11* (five tumours), consistent with somatic variants in these genes being common initiating events in UMs. The variant allele fraction (VAF) of these *GNAQ* and *GNA11* variants indicated their presence in the majority (66–100%) of cells in both Regions A and B of all tumours. We also detected *BAP1* variants in both regions in four tumours, as described above, each with a VAF consistent with their presence in most or all tumour cells (82–100%) and concomitant deletion of the other *BAP1* allele.

We also detected a range of additional variants that are not typically associated with UM (Figure 1 and Table 4). In Case 1, we identified a variant in *ARID1A*, located within chromosome band 1p36, in both regions with a VAF of 0.90 and 0.94 (Table 4), consistent with a concomitant deletion of the other allele. Inspection of the CGH/SNP chromosome microarray data identified a heterozygous deletion in 100% of cells encompassing chromosome bands 1p36.33-p34.3 (37.88 Mb), which contains the *ARID1A* locus. In the RNA from this tumour, we detected a *MET* exon 14 skipping transcript with a total of 1025 *MET* exons 13–15 spanning reads in Region B (0 reads were detected in Region A). Resequencing following RNA extraction without microdissection failed to detect the *MET* exon 14 skipping transcript. Closer inspection of the raw data indicated that *MET* exons 13–15 spanning reads were present; however, the number of reads (413) fell below the limit of detection for the assay (1000 reads). This suggests that the *MET* exon 14 skipping transcript originated from only a subset of cells analysed, which is consistent with the fact that this transcript is present specifically in Region B of this tumour. In Case 2, a variant in *MLH1*, located within chromosome band 3p22, was detected with a high VAF (0.87 and 0.72 in Regions A and B, respectively). CGH/SNP chromosome microarray data showed that, in addition to the heterozygous loss of chromosome 3p (3p26.3-p14.3 (55.28 Mb) in 80–100% of cells), a region including *BAP1* and *MLH1*, this tumour also exhibited the heterozygous loss of chromosome 3q (3q25.32-q29 (35.29 Mb) in 80–100% of cells) and heterozygous loss of chromosome 6q (6q16.2-q27). Although a loss of a copy of MLH1 has been demonstrated in monosomy 3 UM, and variants in PMS2 and MSH3 are extremely rare in UM, a variant in the remaining MLH gene has not been reported [38]. Case 2 also contained an *SF3B1* variant with a VAF of 0.43 in Region A and 0.26 in Region B. No variants that were specific to Region A or Region B were identified. In Case 3, a *CDK12* variant was detected in all cells of Regions A and B; whereas, four variants in *ATRX*, *BRCA2*, *PIK3CA*, and *PPARG* were detected specifically in Region A. All four variants displayed low VAFs (0.05–0.07), indicating a late molecular event present in a small subset of cancer cells. In Case 4, a *TSC1* variant was detected in all cells of Regions A and B (VAF 0.48 and 0.47, respectively), and an *ATRX* variant was detected specifically in Region B with a VAF of 0.06, indicating a late molecular event. This *ATRX* variant was different from that detected in Case 3. Case 5 contained a *NOTCH1* and an *SLX4* variant in all cells of Regions A and B; however, four additional variants specific to Region B were also detected. This included a second variant in *NOTCH1* with a VAF of 0.26, suggesting that it arose early, following the divergence of cells in Region B; whereas, the variants in *FANCD2*, *ATR*, and *PTEN* (VAF 0.07, 0.06, and 0.05, respectively) are late molecular events. In Case 6, no variants that were specific to Region A or Region B were identified; however, a *BRCA2* variant was detected in all cells in both regions. In Case 7, *SMARCB1* and *FANCA* variants were detected in the majority of cells (62–92%) in both regions; however, the VAF of an *ATR* variant (0.28 and 0.13 in Regions A and B, respectively) indicated that this was a later event in the development of this tumour. No variants that were specific to Region A or Region B were identified.

We determined which of the genes identified were listed in the eligibility criteria for existing clinical trials. Variants in 10 of the genes implicated in our cohort of UMs served as eligibility criteria for current phase I/II clinical trials in UM or advanced solid tumours (Table 5).

Importantly, four genes (*MET*, *ATRX*, *ATR*, and *BRCA2*) associated with active clinical trials were mutated specifically in one region of the tumours investigated, suggesting that may have been missed if tumour heterogeneity had not been considered.

### 3.4. Recurrently Mutated Genes in UM

**Figure 2:**
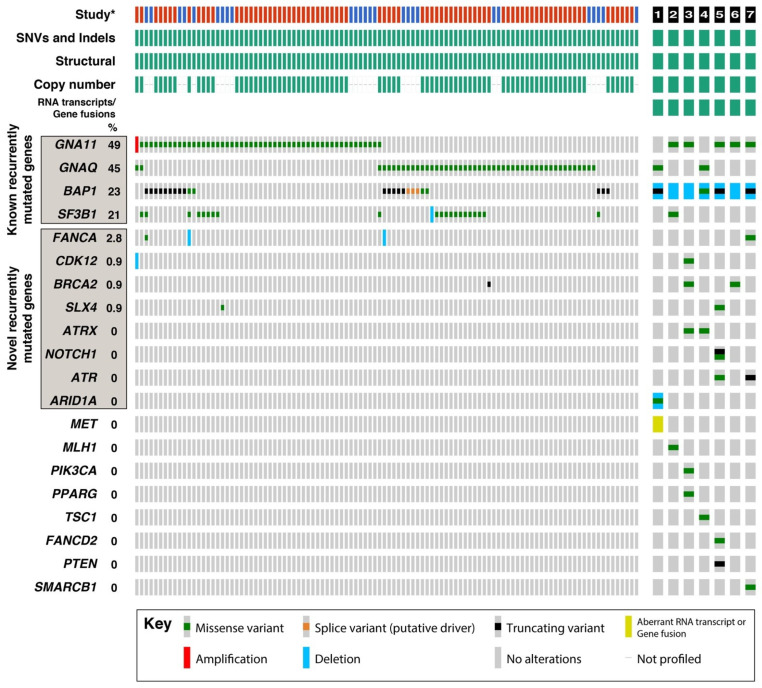
Recurrently mutated genes in UM. Top panel, left: Gene mutations and chromosome copy number changes as identified by * TCGA study [3] (*n* = 80) and Johansson et al. [39] (n = 28). Each column represents a TCGA single case. Top panel, right: Patients 1 to 7 in our study cohort (n = 7). Bottom panel, left: Recurrent and rarer gene mutations identified by TCGA. Bottom panel, right: Mutations identified in our study cohort. In addition to the common UM gene variants, we identified gene variants not previously described in all 7 cases, 3 of which were recurrent. which are indicated by the red, blue, and black boxes, respectively. Mutations include sequence variants (SNVs and indels), inversions and translocations (structural), and copy number alterations (deletions or amplifications). Gene fusions identified by RNA transcripts were not reported by TCGA. Recurrently mutated genes were defined as those showing multiple alterations in these datasets. Percentage values next to each gene symbol represent the frequency of alterations in data from TCGA [3] and Johansson et al. [39]. Genes are listed in order of mutation frequency. Note that deletions involving MLH1 have been omitted, as these are part of chromosome 3 deletions also involving BAP1.

The mining of data from TCGA [3] and Johansson et al. [39] identified 108 UMs profiled for SNVs and indels, copy number alterations, and structural variants. We determined the frequency of these types of genetic alterations in all genes identified as mutated in our study (Figure 2). Five genes (*GNAQ*, *GNA11*, *BAP1*, *SF3B1*, and *FANCA*) were mutated across all three studies. We also identified genes that were mutated once in our cohort of UMs and in at least one other study (CDK12, BRCA2, and SLX4) or multiple times in our cohort of UMs (ATRX, NOTCH1, ATR, and ARID1A). In summary, our data confirmed the importance of known canonical genes but also identified novel recurrently mutated genes in the development of UM.

## 4. Discussion

There is no established cure for metastatic UM; therefore, the identification of potential therapies and clinical trials for these patients offers a rare opportunity to extend disease-free survival. We show that heterogeneity in pigmentation can be used to guide the microdissection and molecular testing of UMs and to identify therapeutically relevant variants that might otherwise be missed due to tumour heterogeneity. The rationale for using heterogeneity in pigmentation as a guide for microdissection is that, in our experience, it is a relatively common and conspicuous finding in larger, enucleated UMs, is readily discerned on macrophotography, and lends itself well to microdissection on corresponding paraffin sections. Since melanin production is a marker of melanocytic differentiation, loss of melanin may indicate tumour progression via dedifferentiation. Enucleation is typically reserved for advanced-stage tumours, too large for plaque brachytherapy; these UMs have a higher risk of metastasis, presenting a compelling opportunity to investigate gene variants associated with metastasis or those potentially amenable to available targeted therapies.

Molecular heterogeneity within primary UMs has been previously described [40,41,42]. Shain et al. [41] documented molecular heterogeneity in 16 of 35 primary UMs and showed that, when separate regions were microdissected for sequencing, additional alterations, such as *BAP1* variants and chromosome 8q gain, were identified. Significantly, variants found in liver metastases matched those found in specific regions of the primary tumour. Our study extends on these findings by showing that molecular heterogeneity can be captured through the analysis of macroscopically distinct regions. We used pigmentation as a visible differential, but other differences across a tumour, such as histological or immunohistochemical differences, may equally serve as adequate markers of molecular heterogeneity. While our findings add to the emerging evidence that UM heterogeneity may influence metastatic behaviour, proof of this concept will require the comparison of the gene variants in matched, primary metastasis tumour pairs.

While tumour heterogeneity is unlikely to be restricted to large UMs, undertaking proof of concept studies in smaller tumours is logistically challenging. While a tissue diagnosis is a core tenet of best practice for managing all UMs, the tumour sample available for patients with smaller tumours undergoing plaque brachytherapy is a small, fine-needle aspiration biopsy. In the event there is sufficient tissue excess for routine diagnostic and prognostic needs, detecting tumour heterogeneity (by single-cell sequencing technologies, for example) cannot overcome the reality that an aspirate is unlikely to be representative of the entire tumour.

Across many different tumour types, including UM, molecular heterogeneity and the accumulation of additional molecular changes are the basis of tumour progression and metastasis [37]. In UM, cellular and molecular heterogeneity within the primary tumour has been linked to a molecular switch that drives metastasis [40]. Early evidence confirms that the mutational signatures of metastatic UM closely match the phylogenetically advanced regions of heterogenous primaries [41]. This heterogeneity suggests that no single targeted therapy will be effective in the treatment of all metastatic UM. Accounting for this heterogeneity may become increasingly important when determining treatment and prognosis. Though preliminary, our findings encourage the ongoing investigation of heterogeneity in primary UM based on morphologically discernible tumour regions, with the eventual goal of identifying additional treatment options for metastatic disease.

Our study presents the two following significant findings: (1) we identified therapeutically relevant variants in our cohort of UMs, and (2) we identified novel, recurrently mutated genes associated with UM development. One of the most intriguing findings in our study was the detection of a *MET* exon 14 skipping transcript in Region B of one tumour. The *MET* exon 14 skipping transcript is an emerging biomarker in metastatic non-small cell lung cancer [43,44,45,46,47] and other solid tumour types [48] that predicts sensitivity to capmatinib, crizotinib, and tepotinib. These targeted therapies are recommended by multiple professional guidelines, including NCCN and ESMO, as treatment options for patients with non-small cell lung cancer that harbour *MET* exon 14 skipping variants. The presence of this biomarker specifically in one region indicates a later event in the development of this tumour. The *MET* (Mesenchymal to Epithelial Transition) gene encodes the hepatocyte growth factor receptor, a receptor tyrosine kinase that is important in embryogenesis, and cellular survival, migration, and invasion. The expression of exon 14 skipping transcripts results in constitutively active kinase activity due to the in-frame exclusion of a regulatory juxtamembrane domain. While *MET* exon 14 skipping transcripts have not previously been reported in primary UMs, there is emerging evidence of cMET protein overexpression in primary and metastatic UM [25], indicating a promising therapeutic target for patients with metastatic disease. Importantly, we identified this biomarker using targeted RNA sequencing, which is currently not performed as part of the routine testing of UMs. Further research utilising RNA sequencing or targeted gene fusion analysis should be performed to determine whether this transcript, or gene fusions, are recurrent events. Finally, we identified four current (at the time of writing) clinical trials for patients with UM and advanced solid tumours with eligibility criteria that included variants in genes that were identified specifically in one region of a tumour in our study. This provides further support for the analysis of multiple regions in heterogeneous tumours for identifying additional relevant clinical trials or possible targeted therapies, such as capmatinib, crizotinib, or tepotinib.

Comparisons with previous studies allowed us to identify eight genes (*FANCA*, *CDK12*, *SLX4*, BRCA2, *ATRX*, *NOTCH1*, *ATR*, and *ARID1A*) that were either not previously described or very rarely reported in UM. Two of these genes (*NOTCH1* and *ARID1A*) were each targeted by two mutational events in two different tumours. The specificity of two mutational events targeting the same gene in a single tumour suggests that they are important in the pathogenesis of that particular tumour; whereas, the identification of genes that are recurrently mutated across a relatively small cohort of UMs implicates them more broadly in UM development. The importance of these novel genes is supported by another recent study that used whole-genome sequencing to identify variants present in an additional 103 UMs [48]. When cases from our study (*n* = 7), TCGA [3] (*n* = 80), Johansson et al. [39] (*n* = 28), and Johansson et al. [48] (*n* = 103) were combined (*n* = 218), four genes were identified as mutated in <2% of UM cases: *FANCA* (1.8%, four cases), *SLX4* (1.8%, four cases), *BRCA2* (1.4%, three cases), and *ATRX* (1.4%, three cases). None of the specific variants identified were common among these studies, and the role of these genes in UM development remains to be determined. However, the established role of *FANCA*, *SLX4*, *BRCA2*, and *ATRX* in DNA repair pathways is unlikely to be coincidental, especially considering that we identified mutations in other DNA repair pathway genes, including *ATR*, which was mutated in two tumours in our study. The association between germline BRCA2 mutations and UM risk requires further investigation; earlier literature suggests less than 2% of UMs is attributable to germline BRCA2 variants [49,50,51].

In three tumours, our analysis failed to identify variants that were specific to Region A or Region B of the tumour. We attribute this to the possible presence of variants in genes that were not targeted in our study. Specifically, our analysis did not target the other known driver genes involved in UM (*PLCB4*, *CYSLTR2*, and *EIF1AX*) or significantly mutated genes that have recently been identified, such as *TP53BP1*, *CSMD1*, *TTC28*, *DLK2*, *KTN1*, *RPL5*, and *CENPE* [2,48]. Nevertheless, a strength of our study is the depth of the sequencing performed (mean coverage depth ≥ 1000×) in different regions of the same tumour, which enabled us to identify several novel genes not identified in previous studies.

## 5. Conclusions

Our observations support the approach of separately sequencing distinct morphological regions within larger UMs to better identify therapeutically relevant mutations. This includes the identification of existing targeted therapies that are approved in other tumour types or the identification of relevant clinical trials. Our observations require confirmation in a larger case series and prospective studies. This may include analysis using other platforms, including transcriptome sequencing, to further characterise differences in the mutational and transcriptional landscapes of UM.

## Figures and Tables

**Table 1:** Patient and tumour characteristics.

Patient and Tumour Characteristics		*n*/years
Age at enucleation (years)	Range	40–82
	Average	59.3
Sex	Male	6
	Female	1
Patient status	Alive	5
	Deceased	2
Affected eye	Left	4
	Right	3
Ciliary body involvement	Yes	6
	No	1
Tumour thickness	<12 mm	6
	12–15 mm	1
Tumour basal diameter	<15 mm	1
	15–18 mm	5
	>18 mm	1
Extraocular extension	Absent	7
	Present	0
pTNM stage	pT2	0
	pT3a	0
	pT3b	5
	pT4a	1
	pT4b	1
Metastasis	Present	3
	Absent	4

pTNM stage was determined according to AJCC 2017 criteria [10].

**Table 2:** Histopathological characteristics of Regions A and B in each UM.

Case #	1		2		3		4		5		6		7	
Region	A	B	A	B	A	B	A	B	A	B	A	B	A	B
Tumour pigmentation	+++	−	+++	++	+	−	+	−	+++	+	+++	++	+++	+
Tumour growth pattern	Solid	Solid	Solid and diffuse	Solid	Solid	Solid	Solid	Solid	Solid	Solid	Solid	Solid	Solid & diffuse	Solid
Cell type	Mixed	Mixed	Mixed	Mixed	Mixed	Mixed	Mixed	Mixed	Mixed	Mixed	Mostly spindled	Mostly spindled	Epithelioid	Epithelioid
Mitosis per 40 HPF	21	ND	23	ND	23	ND	23	ND	12	ND	18	ND	19	ND
Microvascular density ^1^	Low	Low	High	Mod	Mod	Low	High	Mod	Low	Very low	High	High	Very high	High
Extravascular matrix pattern	Loops	LFN	Loops absent	Loops	LFN	LFN	LFN	LFN	LFN	Loops	LFN	LFN	Loops	LFN
Vascular lakes	Present	Present	Present	Present	Present	Present	Present	Present	Present	Absent	Present	Present	Absent	Present
Infiltrating lymphocytes	Low	Low	Low	Low	High	High	High	High	Low	High	Low	Low	High	Low
Infiltrating macrophages	CD68+ High CD163+ Very high	CD68+ Mod CD163+ Mod	CD68+ High CD163+ Low	CD68+ Low CD163+ Low	CD68+ Low CD163+ High	CD68+ Low CD163+ Mod	CD68+ High CD163+ High	CD68+ Very high CD163+ Very high	CD68+ Low CD163+ Low	CD68+ Low CD163+ Mod	CD68+ Mod CD163+ High	CD68+ Mod CD163+ High	CD68+ High CD163+ Very high	CD68+ Low CD163+ High

Histopathologic features the cohort. The two distinct morphological regions in each tumour (1 to 7; top row) are identied as A and B (second row). +++ Heavily pigmented. ++ Moderately pigmented. + Lightly pigmented. − Amelanotic. ND, not determined. ^1^. Microvascular density by CD31 immunohistochemistry. LFN = loop-forming networks, Mod = moderate. Grey shading indicates tumour regions with highest pigmentation.

**Table 3:** High metastatic risk features in all tumours examined.

	Case #						
	1	2	3	4	5	6	7
BAP1 IHC	Aberrant	Aberrant	Aberrant	Aberrant	Aberrant	Aberrant	Aberrant
Chromosome 3	Monosomy 3	Loss of 3p (1 copy) *	Monosomy 3	Monosomy 3	Monosomy 3	Monosomy 3	Loss of 3p (1 copy) *
Chromosome 8q	Gain (5 copies)	Disomy	Gain (3 copies)	Gain (4–5 copies)	Gain (3 copies)	Disomy	Gain (3 copies)
BAP1 sequence variants	Detected c.1766_1770 del	Not detected	Not detected	Detected c.518A>G	Detected c.172_179del	Not detected	Detected c.1153C>T

High metastatic risk features are defined as loss of monosomy 3 and chromosome 8q gain. CGH/SNP chromosome microarray and *BAP1* sequence variants, identified using targeted sequencing. *BAP1* variants are described relative to RefSeq transcript NM_004656.4. * Deletions encompass the *BAP1* locus on 3p.

**Table 4:** Sequence variants identified by targeted sequencing in Regions A and B in each tumour.

Case #	Gene and Variant (HGVS)	Region A VAF	Region B VAF	gnomAD AF (Allele Count [Homozygotes])	AMP/ASCO/CAP Tier	Gene Implicated Previously in UM
1	NM_002072.4(*GNAQ*):c.626A>T (p.Gln209Leu)	0.51	0.48	Not present	Tier 2C	Yes [3,34,37]
	NM_006015.5(*ARID1A*):c.4550C>G (p.Ala1517Gly)	0.90	0.94	Not present	Tier 2C	No
	NM_004656.4(*BAP1*):c.1766_1770del (p.Ile589Argfs*52)	0.80	0.81	Not present	Tier 2C	Yes [3,34,37]
	NM_001127500.2:*MET* exon 14 skipping transcript variant ^Δ^	ND	Detected	NA	Tier 2C	No
2	NM_002067.4(*GNA11*):c.626A>T (p.Gln209Leu)	0.44	0.33	Not present	Tier 2C	Yes [3,34,37]
	NM_012433.3(*SF3B1*):c.1873C>T (p.Arg625Cys)	0.43	0.26	Not present	Tier 2C	Yes [3,34,37]
	NM_000249.3(*MLH1*):c.1752C>G (p.Asp584Glu)	0.87	0.72	Not present	Tier 3	No
3	NM_002067.4(*GNA11*):c.626A>T (p.Gln209Leu)	0.48	0.40	Not present	Tier 2C	Yes [3,34,37]
	NM_016507.3(*CDK12*):c.1339T>A (p.Leu447Ile)	0.51	0.51	0.0001315 (20[0])	Tier 3	Yes [3]
	NM_000059.3(*BRCA2*):c.3812C>T (p.Ser1271Leu)	0.06	ND	Not present	Tier 2C	Yes, [3]
	NM_000489.5(*ATRX*):c.4525G>A (p.Glu1509Lys)	0.07	ND	Not present	Tier 2C	Yes [37]
	NM_006218.3(*PIK3CA*):c.1714G>A (p.Val572Ile)	0.06	ND	Not present	Tier 3	No
	NM_138712.3(*PPARG*):c.392C>T (p.Ser131Cys)	0.05	ND	Not present	Tier 3	No
4	NM_002072.4(*GNAQ*):c.626A>C (p.Gln209Pro)	0.46	0.54	Not present	Tier 2C	Yes [3,37]
	NM_004656.4(*BAP1*):c.518A>G (p.Tyr173Cys)	0.86	0.76	Not present	Tier 2C	Yes [3,37]
	NM_000368.4(*TSC1*):c.727G>T (p.Asp243Tyr)	0.48	0.47	Not present	Tier 3	No
	NM_000489.5(*ATRX*):c.6677G>A (p.Arg2226Lys)	ND	0.06	Not present	Tier 2C	Yes [37]
5	NM_002067.4(*GNA11*):c.626A>T (p.Gln209Leu)	0.33	0.39	Not present	Tier 2C	Yes
	NM_004656.4(*BAP1*):c.172_179del (p.Ser58Lysfs*8)	0.41	0.78	Not present	Tier 2C	Yes
	NM_017617.5(*NOTCH1*):c.2636G>A (p.Arg879Gln)	0.44	0.52	0.0001051 (16[0])	Tier 3	Yes [37]
	NM_032444.3(*SLX4*):c.506C>T (p.Ser169Leu)	0.36	0.36	Not present	Tier 2C	Yes [34,37]
	NM_017617.5(*NOTCH1*):c.4129_4130insT (p.Pro1377Leufs*28)	ND	0.26	Not present	Tier 3	Yes [37]
	NM_033084.4(*FANCD2*):c.2888C>T (p.Pro963Leu)	ND	0.07	Not present	Tier 3	No
	NM_001184.3(*ATR*):c.4454G>A (p.Ser1485Asn)	ND	0.06	Not present	Tier 2C	No
	NM_000314.7(*PTEN*):c.511C>T (p.Gln171*)	ND	0.05	Not present	Tier 3	No
6	NM_002067.4(*GNA11*):c.626A>T (p.Gln209Leu)	0.46	0.42	Not present	Tier 2C	Yes [3,34,37]
	NM_000059.3(*BRCA2*):c.2803G>A (p.Asp935Asn)	0.48	0.50	0.0006506 (99[0])	Tier 2C	Yes [3]
7	NM_004656.4(*BAP1*):c.1153C>T (p.Arg385*)	0.64	0.75	Not present	Tier 2C	Yes [3,34,37]
	NM_002067.4(*GNA11*):c.547C>T (p.Arg183Cys)	0.41	0.44	Not present	Tier 2C	Yes [3,34,37]
	NM_003073.4(*SMARCB1*):c.1130G>A (p.Arg377His)	0.36	0.44	0.000006568 (1[0])	Tier 3	No
	NM_000135.4(*FANCA*):c.480G>A (p.Met160Ile)	0.46	0.42	0.0002891 (44[0])	Tier 2C	Yes [3,34,37]
	NM_001184.3(*ATR*):c.7792C>T (p.Arg2598*)	0.28	0.13	Not present	Tier 2C	No

Variants annotated according to Human Genome Variation Society (HGVS) nomenclature. Variants curated based on potential therapeutic, diagnostic, or prognostic significance according to standards and guidelines from the Association for Molecular Pathology, American Society of Clinical Oncology, and College of American Pathologists (AMP/ASCO/CAP) [36]. Note that the AMP/ASCO/CAP tier provided may change over time as evidence and literature accumulates. AF (allele frequency) was obtained using The Genome Aggregation Database (gnomAD, v3.1.2). VAF, variant allele fraction. ND, not detected. NA, not applicable. UM, uveal melanoma. ^Δ^ The specific DNA variant that results in this transcript (e.g., exon 14 deletion or splicing variant) was not identified. Grey shading indicates region-specific variants.

**Table 5:** Mutated genes and associated clinical trials.

Clinical Trials (Phase)	Tumour Type	Gene(s)
NCT03947385 (Phase I/II)	UM	*GNAQ*, *GNA11*
NCT02693535 (Phase II)	Advanced solid tumours	*MET* exon 14 skipping transcript *
NCT03207347 (Phase I/II)	UM	*ARID1A*, ATR *, *BAP1*, PTEN *, *SLX4*
NCT03767075 (Phase II)	Advanced solid tumours	DNA repair genes (*ATRX* *, ATR *, *BAP1*, *BRCA2* *, *SLX4*, *ARID1A*)
NCT01971515 (Phase I)	Advanced malignancies	*PIK3CA* *, *PTEN* *, *TSC1*
NCT02961283 (Phase I)	Advanced solid tumours	*PIK3CA* *, *PTEN* *
NCT04171700 (Phase II)	Advanced solid tumours	*BRCA2* *, *FANCA*

* Indicates genes with variants identified specifically in one region of a tumour investigated in this study. UM, uveal melanoma. Clinical trial information was obtained from Clinical Trials.gov. Eligibility dependent on inclusion and exclusion criteria for each trial. Grey shading indicates trials that were active and recruiting at time of writing.

## Data Availability

Raw data for this study were generated at the Kinghorn Centre for Clinical Genomics Sequencing Laboratory, Garvan Institute of Medical Research. The data supporting the findings of this study are available in the NCBI Gene Expression Omnibus repository (https://www.ncbi.nlm.nih.gov/geo/ (accessed on 9 November 2023)).

## Supplementary Materials

The following supporting information can be downloaded at: https://www.mdpi.com/article/10.3390/cancers16244152/s1, Figure S1: Tumour microdissection. Table S1: The primary antibodies, antigen retrieval, and incubation time are summarised. Tables S2 and S3: a full description of the targeted genes and gene fusion events.
