# Supplementary material for: Microdissection of Distinct Morphological Regions Within Uveal Melanomas Identifies Novel Drug Targets
Source: Cancers (Basel). 2024 Dec 13;16(24):4152. doi: 10.3390/cancers16244152 (PMC11674814; doi:10.3390/cancers16244152)
Supplement: Supplementary file 1 [file cancers-16-04152-s001.zip › Supplementary Figure 1 caption.pdf]

Supplementary Figure 1: Caption

(a) Ciliochoroidal uveal melanoma with two macroscopically distinct appearing regions. A is a highly pigmented region and B is a less pigmented region. (b) The H&E section of the same tumour with corresponding areas A and B marked after microscopic assessment and used to guide the microdissection of the unstained paraffin section (c) to allow each tumour region to be sequenced separately.
