# Supplementary material for: Microdissection of Distinct Morphological Regions Within Uveal Melanomas Identifies Novel Drug Targets
Source: Cancers (Basel). 2024 Dec 13;16(24):4152. doi: 10.3390/cancers16244152 (PMC11674814; doi:10.3390/cancers16244152)
Supplement: Supplementary file 1 [file cancers-16-04152-s001.zip › Table S1. Primary antibodies.pdf]

Table S1. Immunohistochemistry- primary antibodies.

**Antibody**	**Supplier**	**Category**	**Pre-treatment procedure**	**Antibody incubation (min)**	**Dilution**
**CD163 red**	Leica Novocastra	NCL-CD163	HIER retrieval 36min at 95C	16	1/100
**CD68 (P) red**	DAKO	M0876	Enzyme retrieval Protease1 8mins	32	1/50
**MART**	Cell Marque	281M-96	HIER retrieval 36min at 95C	16	1/100
**CD31**	Sigma	HPA045153	HIER retrieval 36min at 95C	60	1/50
**BAP1**	Santa Cruz	sc-28383	HIER retrieval 64min at 95C	32	1/50
