# Supplementary material for: Microdissection of Distinct Morphological Regions Within Uveal Melanomas Identifies Novel Drug Targets
Source: Cancers (Basel). 2024 Dec 13;16(24):4152. doi: 10.3390/cancers16244152 (PMC11674814; doi:10.3390/cancers16244152)
Supplement: Supplementary file 1 [file cancers-16-04152-s001.zip › Table S2 Oncomine Comprehensive Assay v3.pdf]

Supplementary Table S2: Oncomine Comprehensive Assay v3 Genes and Gene Fusions

**Hotspot Genes**				**Full-Length Genes**		**Copy Number Genes**		**Gene Fusions (Inter-and Intra-genetic**	
AKT1	*FGFR4*	*MED12*	*SRC*	*ARID1A*	*PALB2*	*AKT1*	*MDM2*	*AKT2*	*NF1*
AKT2	*FLT3*	*MET*	*STAT3*	*ATM*	*PIK3R1*	*AKT2*	*MDM4*	*ALK*	*NOTCH1*
AKT3	*FOXL2*	*MTOR*	*TERT*	*ATR*	*PMS2*	*AKT3*	*MET*	*AR*	*NOTCH4*
ALK	*GATA2*	*MYC*	*TOP1*	*ATRX*	*POLE*	*ALK*	*MYC*	*AXL*	*NRG1*
AR	*GNA11*	*MYCN*	*U2AF1*	*BAP1*	*PTCH1*	*AR*	*MYCL*	*BRAF*	*NTRK1*
ARAF	*GNAQ*	*MYD88*	*XPO1*	*BRCA1*	*PTEN*	*AXL*	*MYCN*	*BRCA1*	*NTRK2*
AXL	*GNAS*	*NFE2L2*		*BRCA2*	*RAD50*	*BRAF*	*NTRK1*	*BRCA2*	*NTRK3*
BRAF	*H3F3A*	*NRAS*		*CDK12*	*RAD51*	*CCND1*	*NTRK2*	*CDKN2A*	*NUTM1*
BTK	*HIST1H3B*	*NTRK1*		*CDKN1B*	*RAD51B*	*CCND2*	*NTRK3*	*EGFR*	*PDGFRA*
CBL	*HNF1A*	*NTRK2*		*CDKN2A*	*RAD51C*	*CCND3*	*PDGFRA*	*ERBB2*	*PDGFRB*
CCND1	*HRAS*	*NTRK3*		*CDKN2B*	*RAD51D*	*CCNE1*	*PDGFRB*	*ERBB4*	*PIK3CA*
CDK4	*IDH1*	*PDGFRA*		*CHEK1*	*RB1*	*CDK2*	*PIK3CA*	*ERG*	*PPARG*
CDK6	*IDH2*	*PDGFRB*		*CREBBP*	*RNF43*	*CDK4*	*PIK3CB*	*ESR1*	*PRKACA*
CHEK2	*JAK1*	*PIK3CA*		*FANCA*	*SETD2*	*CDK6*	*PPARG*	*ETV1*	*PRKACB*
CSF1R	*JAK2*	*PIK3CB*		*FANCD2*	*SLX4*	*EGFR*	*RICTOR*	*ETV4*	*PTEN*
CTNNB1	*JAK3*	*PPP2R1A*		*FANCI*	*SMARCA4*	*ERBB2*	*TERT*	*ETV5*	*RAD51B*
DDR2	*KDR*	*PTPN11*		*FBXW7*	*SMARCB1*	*ESR1*		*FGFR1*	*RAF1*
EGFR	*KIT*	*RAC1*		*MLH1*	*STK11*	*FGF19*		*FGFR2*	*RB1*
ERBB2	*KNSTRN*	*RAF1*		*MRE11A*	*TP53*	*FGF3*		*FGFR3*	*RELA*
ERBB3	*KRAS*	*RET*		*MSH2*	*TSC1*	*FGFR1*		*FGR*	*RET*
ERBB4	*MAGOH*	*RHEB*		*MSH6*	*TSC2*	*FGFR2*		*FLT3*	*ROS1*
ERCC2	*MAP2K1*	*RHOA*		*NBN*		*FGFR3*		*JAK2*	*RSPO2*
ESR1	*MAP2K2*	*ROS1*		*NF1*		*FGFR4*		*KRAS*	*RSPO3*

EZH2	*MAP2K4*	*SF3B1*		*NF2*		*FLT3*		*MDM4*	*TERT*
FGFR1	*MAPK1*	*SMAD4*		*NOTCH1*		*IGF1R*		*MET*	
FGFR2	*MAX*	*SMO*		*NOTCH2*		*KIT*		*MYB*	
FGFR3	*MDM4*	*SPOP*		*NOTCH3*		*KRAS*		*MYBL1*	
