# Supplementary figures and images for: Microdissection of Distinct Morphological Regions Within Uveal Melanomas Identifies Novel Drug Targets
Source: Cancers (Basel). 2024 Dec 13;16(24):4152. doi: 10.3390/cancers16244152 (PMC11674814; doi:10.3390/cancers16244152)

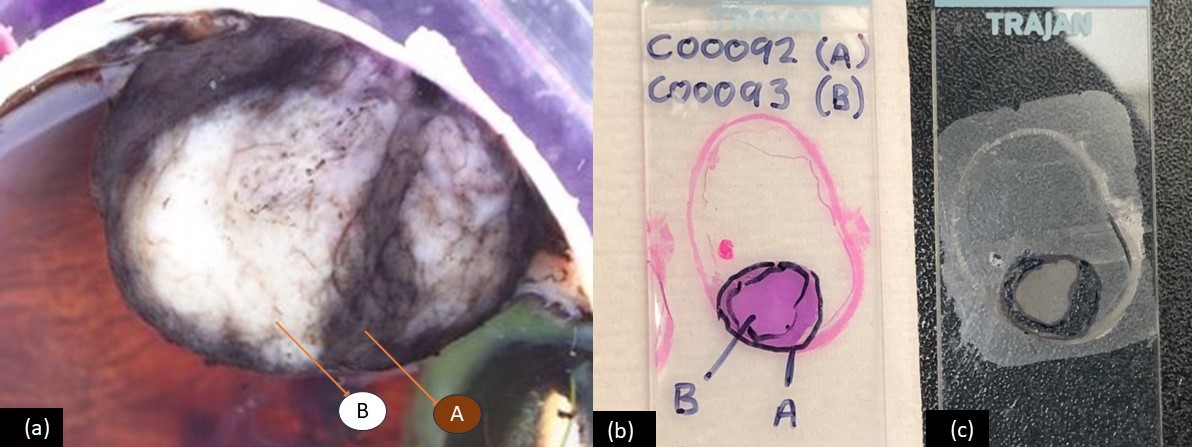

Supplement: Supplementary file 1 [file cancers-16-04152-s001.zip › Supplementary fig 1.jpg]
